# Cryodevices developed for minimum volume cooling vitrification of bovine oocytes

**DOI:** 10.1111/asj.13683

**Published:** 2022-01-24

**Authors:** Shinichi Hochi

**Affiliations:** ^1^ Faculty of Textile Science and Technology Shinshu University Ueda Nagano Japan

**Keywords:** bovine oocytes, cryodevice, mass batch, MVC vitrification, water absorption

## Abstract

Unfertilized bovine oocytes can be efficiently cryopreserved only when an extremely rapid cooling rate (>20,000°C/min) is applied to oocytes with a very limited amount of surrounding vitrification solution. This protocol is defined as minimum volume cooling (MVC) vitrification. Various types of cryodevices, such as open pulled straw, Cryoloop, and Cryotop, have been developed to accelerate the cooling efficacy. Furthermore, hollow fibers with nano‐scale pores, triangle nylon mesh sheets, and multilayer silk fibroin sheets have been optimized for the loading of large quantities of oocytes and/or the subsequent removal of excess vitrification solution, without requiring skillful operation to transfer individual oocytes using fine capillaries. This article provides an up‐to‐date review of cryodevices suitable for the MVC vitrification of bovine oocytes at the immature (germinal vesicle‐) and mature (metaphase II‐) stages.

## 1 INTRODUCTION

Meiosis‐arrested bovine oocytes are very large spherical cells (diameter = 140 μm, enclosed with an acellular zona pellucida) surrounded by cumulus cell layers after ovulation and prior to sperm penetration. Cryopreserved oocytes provide a valuable resource for embryo production via assisted reproductive technology (ART), including in vitro fertilization (IVF), intracytoplasmic sperm injection, or somatic cell nuclear transfer in domestic animal industries. However, retrieval of bovine oocytes after cryopreservation is not fully sufficient for subsequent embryo production. Likely obstacles for successful cryopreservation of bovine oocytes included the high volume/surface ratio, depolymerization of spindle tubulin, abnormal aster formation, and premature release of cortical granules of metaphase‐II stage (MII) oocytes (Hwang & Hochi, 2014). In addition, oocytes from domestic species, such as cattle and pigs, are known to be highly sensitive to cryoinjuries due to the high level of cytoplasmic lipid droplets when compared with human and rodent oocytes (Zhou & Li, 2013). L‐carnitine has been used to reduce intracellular lipids and improve the cryotolerance of bovine MII oocytes (Chankitisakul et al., 2013; Sprícigo et al., 2017). During the past decade, anti‐oxidative chemicals, such as α‐tocopherol (Yashiro et al., 2015), melatonin (Zhao et al., 2016), and resveratrol (Chinen et al., 2020; Sprícigo et al., 2017), were also applied to improve oocyte cryotolerance.

Vitrification (Rall & Fahy, 1985), as a replacement for conventional freezing (Whittingham et al., 1972; Wilmut & Rowson, 1973), offered a simple, cost‐effective, and efficient protocol in embryo cryopreservation, by the formation of an amorphous glass state rather than detrimental ice crystals. This protocol includes the dehydration of embryos by exposure to a concentrated vitrification solution (VS), followed by direct immersion of 0.25‐ml plastic straw containers into liquid nitrogen (LN_2_; 2,000–2,500°C/min), rather than dehydration during a slow cooling process in a conventional freezing regimen (0.3–2°C/min). Technicians are familiar with using plastic straw for embryo vitrification, because plastic straws have been used for bovine semen freezing/artificial insemination (0.5‐ml volume) and embryo slow freezing/transplantation (0.25‐ml volume). Traditional straw vitrification does not require specialized freezing equipment for a constant rate of slow cooling, but the time allowed for exposure to highly toxic VS is quite short (approximately 1 min), resulting in a tight time schedule and a decreased sample number per single operation, as well as increased pressure upon technicians. The use of straw vitrification for bovine oocytes remained inefficient, despite of a 9% blastocyst yield (calculated from the total number of cryopreserved oocytes unless specified hereafter) and the in vivo development to two fetuses from post‐warmed oocytes reported in an early study (Hamano et al., 1992). This article provides an up‐to‐date review of cryodevices suitable for minimum volume cooling (MVC) vitrification of bovine oocytes at the immature germinal vesicle (GV)‐ and mature MII‐stages.

## 2 CRYODEVICES FOR OOCYTE MVC VITRIFICATION

**FIGURE 1:**
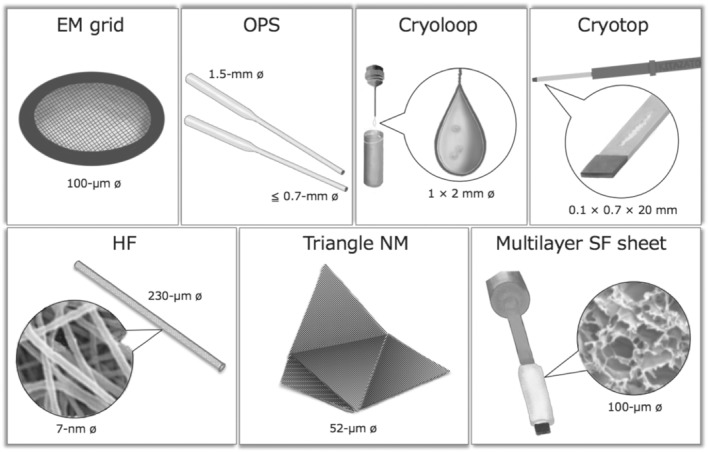
Cryodevices used for oocyte MVC vitrification. Tubing‐type: open pulled straw (OPS) and hollow fiber (HF). Surface‐type: electron microscope (EM) grid, Cryoloop, Cryotop, triangle nylon mesh (NM), and multilayer silk fibroin (SF) sheet

A significant breakthrough for the vitrification of bovine oocytes was achieved by the MVC procedure. Typical cryodevices designed for the MVC vitrification of bovine oocytes are illustrated in Figure 1. A 15% blastocyst yield was resulted from post‐warmed MII oocytes vitrified with a MVC protocol using an electron microscope (EM) copper grid as the cryodevice (Martino et al., 1996). The EM grid was originally adopted to cryopreserve chilling‐sensitive *Drosophila melanogaster* embryos, which had a mm‐scale diameter (Mazur et al., 1992; Steponkus et al., 1990). The MVC vitrification protocol involved a 3–15 min exposure of oocytes to equilibration solution containing moderate concentrations of membrane‐permeating cryoprotective agents (CPAs; dimethylsulfoxide [DMSO] and/or ethylene glycol [EG]; 3–20%) and a 1 min exposure to VS containing higher concentrations of the permeable CPAs (30–40%) and a non‐permeating disaccharide (sucrose or trehalose) before rapid cooling to LN_2_ temperature. Within the short exposure time to the VS, oocytes must be placed into or onto a cryodevice with a minimal volume of VS to accelerate the cooling rate. There is no strict definition of the “minimum” volume for bovine oocyte vitrification, but it is generally considered to be less than 1 μl. After storage in LN_2_ and rapid warming, CPAs are removed from the oocytes in a stepwise manner using decreasing concentrations of sucrose solution. Various types of cryodevices have been developed to accelerate the cooling rate of bovine oocytes and can be theoretically divided into two categories: tubing and surface devices (Saragusty & Arav, 2011). Completely device‐less protocols have also been applied to the MVC vitrification of bovine mature oocytes, with a blastocyst yield of 9% in a solid surface vitrification (SSV) system (Dinnyés et al., 2000) and 30% in microdrop (MD) method (Papis et al., 2000). A brief list of blastocyst yields from vitrified‐warmed bovine MII oocytes using different crydevices is shown in Table 1.

**TABLE 1:** MVC vitrification of bovine mature (MII) oocytes using different cryodevices and blastocyst generation following IVF

Cryodevice (tubing [T]/surface [S])	CPA in VS	Blastocyst yield (%)^a^	Reference
Straw [T]	DMSO + PG + AA	9	Hamano et al. (1992)
EM grid [S]	EG + Suc	15	Martino et al. (1996)
OPS [T]	DMSO + EG + Suc	13	Vajta et al. (1998)
SSV [device‐less]	EG + PVP + Tre	9	Dinnyés et al. (2000)
MD [device‐less]	EG + Suc	30	Papis et al. (2000)
Cryotop [S]	EG + PG + Suc	8	Chian et al. (2004)
Cryoloop [S]	DMSO + EG + Suc	11	Checura and Seidel (2007)
Tracing paper [S]	DMSO + EG + Suc	8	Paul et al. (2018)
NM [S]	DMSO + EG + Suc	35	Chinen et al. (2019)
HF [T]	EG + Suc	23	Kornienko et al. (2020)
SF [S]	DMSO + EG + Suc	25	Nakayama et al. (2020)

Abbreviations: AA, acetamide; CPA, cryoprotective agent; DMSO, dimethylsulfoxide; EG, ethylene glycol; EM grid, electron microscope grid; Gal, galactose; HF, hollow fiber; MD, microdrop; NM, nylon mesh; OPS, open pulled straw; PG, propylene glycol; PVP, polyvinyl‐pyrrolidone; SF, silk fibroin; SSV, solid surface vitrification; Suc, sucrose; Tre, trehalose; VS, vitrification solution.

^a^ Blastocyst yields were calculated from the total number of cryopreserved oocytes.

Tubing device: Oocytes are aspirated into a tubing‐type cryodevice. Vajta et al. (1998) reported that a very high cooling rate (>20,000°C/min) can be achieved in an open pulled straw (OPS), resulting in a 13% blastocyst yield from post‐warmed bovine MII oocytes and a calf birth, followed by a few minor modifications as superfine OPS (Isachenko et al., 2001) and sealed pulled straw (Chen et al., 2001). Conventional disposable plastic tools were successfully used for the MVC vitrification of bovine oocytes, as reported with the gel‐loading tip (Tominaga et al., 2005) or flexipet‐denuding pipette (Morató et al., 2008). Because of the big market for human ART, a closed system using CryoTip® (Fujifilm Irvine Scientific) has been commercialized and is widespread in infertility clinics (Kuwayama, Vajta, Ieda, et al., 2005; VerMilyea & Brewer, 2017). Sanitary cryopreservation by the closed system is desired to avoid the possible cross‐contamination of vitrified oocytes with pathogens within the LN_2_ tank, but its importance may be dependent of the target animal species. Recently, hollow fiber vitrification (HFV) has been applied to bovine mature oocytes, with a 23% blastocyst yield from post‐warmed oocytes (Kornienko et al., 2020). The triacetate cellulose hollow fibers (HF; 200‐μm inner diameter, 15‐μm‐thick) used in the above study are enriched with 7‐nm pores in the walls. The HFV system is attractive in terms of increased sample number per device and medium exchange without aspiration‐based collection of the oocytes (Maehara et al., 2012; Matsunari et al., 2012).

Surface device: Oocytes are placed on the surface of a cryodevice with a minimal volume of VS. The presence of excess VS around oocytes adversely affects the ultra‐rapid cooling rate when oocytes are immersed into LN_2_. In addition to the classical EM grid (3‐mm diameter, 37‐μm‐thick, 100‐μm diagonal diameter of square; Martino et al., 1996), several surface‐type cryodevices, including Cryoloop (Checura & Seidel, 2007; Lane & Gardner, 2001; Mavrides & Morroll, 2002), hemi‐straw (Liebermann & Tucker, 2002), and Cryotop (Chian et al., 2004; Kuwayama, Vajta, Kato, et al., 2005), have been reported for the MVC vitrification of mammalian oocytes (more derivatives; Cryoleaf, Cryolock, Vitri‐Inga, and stainless MVAC). Among them, Cryotop combined with a hermetically protective container (Cryotop®; Kitazato Corporation, Shizuoka, Japan) is likely the most convenient surface‐type cryodevice for oocytes/embryos from both domestic animals and humans (Kuwayama, 2007). Cooling and warming rates in Cryotop vitrification were estimated as 69,000 and 118,000°C/min, respectively (Mazur & Seki, 2011). It should not be technically difficult for well‐trained lab technicians (who easily handle oocytes with capillary pipetting) to collect 10–12 oocytes floating in VS and place them onto the surface of a polystyrene or polypropylene strip (0.1 × 0.7 × 20 mm) of the device within 1 min. In our laboratory, standard Cryotop vitrification of bovine MII oocytes resulted in blastocyst yields of 13–16% using 1‐day stored ovaries (Hara et al., 2014; Hwang et al., 2013) and 19–32% using fresh ovaries (Chinen et al., 2019, 2020; Nakayama et al., 2020; Yashiro et al., 2015). Blastocyst yields were further improved by a short‐term recovery culture of post‐warmed oocytes in the presence of a Rho‐associated coiled‐coil kinase inhibitor (Y‐27632, blastocyst yield 18%; Hwang et al., 2013), α‐tocopherol (35%, Yashiro et al., 2015), and resveratrol (39%, Chinen et al., 2020).

## 3 SMART DEVICES FOR BULK OOCYTES AND SELF‐VS ABSORPTION

Oocyte numbers loaded per cryodevice are limited because of the strict requirement in minimizing the VS volume in a tight timetable (recommended quantity 10, possible upper limit 20, in MVC vitrification). Even in the HFV system, the maximum number per single HF was reported to be 12–17 in bovine oocytes (Kornienko et al., 2020) and 20 and 40 in porcine and murine oocytes, respectively (Matsunari et al., 2012). Such a limitation does not cause inconvenience in the clinical cryopreservation of human oocytes, because one to three oocytes are routinely loaded per cryodevice. For bovine species, all oocytes retrieved from a single donor (a pair of ovaries) may be cryopreserved in a single operation if oocyte numbers loaded per cryodevice can be increased. Matsumoto et al. (2001) first reported that as many as 65 bovine immature oocytes can be vitrified‐warmed in bulk using nylon mesh (NM) as a cryodevice. Although no blastocysts were obtained in this early study, the same group achieved an 8% blastocyst yield and a live calf from post‐warmed immature oocytes after a few modifications of the equilibration treatment (Abe et al., 2005).

Our laboratory designed a triangle NM sheet (52‐μm diagonal diameter of square) folded one fourth to generate a developed figure of triangular pyramid, originally for the vitrification of large quantities of pancreatic islets (Yamanaka et al., 2017). By applying the triangle NM device, more than 40 bovine mature oocytes were vitrified‐warmed in bulk, resulting in a cooling rate of 29,000°C/min, a warming rate of 81,000°C/min, and a blastocyst yield of 31% (Chinen et al., 2019). This NM device also allowed the removal of excess VS volume before immersion into LN_2_ and facilitated the CPA dilution process without skillful capillary pipetting; sterilized paper towel placed beneath the NM device absorbed the excess VS and sucrose diluents. Post‐warming treatment with resveratrol further rescued vitrified oocytes on the NM device (blastocyst yield 42%; Chinen et al., 2020). Direct LN_2_ immersion of an NM device with a smaller pore size compared with larger pore size counterparts (52 μm vs. 81 or 109 μm) resulted in a comparable cooling rate but a faster warming rate and higher blastocyst yield from post‐warmed bovine oocytes (Chinen et al., 2019). As Seki and Mazur (2008, 2009) first noted in the MVC vitrification of mouse oocytes, more attention should be paid to the acceleration of the warming rate, rather than the cooling rate, to improve cryosurvival. Minimized volume of the VS surrounding the oocytes on a cryodevice with high thermal conductivity would be associated with the accelerated warming rate.

The minimized volume of the VS may be largely dependent on the skill of the technician and/or the type of cryodevice. Silk fibroin (SF), a biocompatible structural protein extracted from the cocoon of the silkworm *Bombyx mori*, can be easily processed into multiporous hydrogels, films, or sponges (Altman et al., 2003) and has been used as scaffold in the field of tissue engineering or regenerative medicine (Davis et al., 2012; Kasoju & Bora, 2012). For example, a wet sheet fabricated from 1.5% SF solution was wound five times around the plastic strip of a Cryotop device and then air‐dried (Nakayama et al., 2020). A few microliters of VS were absorbed into the multilayer SF sheet within a few seconds, leaving 10–12 bovine oocytes on the surface containing numerous 100‐μm‐scale pores. After multilayer SF sheet vitrification, 25% of the post‐warmed oocytes developed into blastocysts, comparable with the blastocyst yields (22–25%) after Cryotop vitrification and triangle NM vitrification. A similar strategy used a high‐absorption material (membrane filter) attached to the plastic tip of a surface‐type cryodevice, described as the Kitasato Vitrification System (KVS) for the MVC vitrification of mouse embryos (Momozawa et al., 2017, 2019). Tracing paper was also used as the VS‐absorbable cryodevice in the MVC vitrification of bovine oocytes (Paul et al., 2018). Further improvement of the high absorption material and holding tool would help its practical application, such as the commercial KVS product Diamour (Mitsubishi Paper Mills Ltd, Tokyo, Japan).

## 4 APPLICATION TO IMMATURE OOCYTES

Research for the cryopreservation of immature oocytes is important because of the increasing demands to retrieve human immature oocytes in cases of ovarian hyper‐stimulation syndrome or cancer in young patients (Yamanaka et al., 2007). In cattle, the ovum pick‐up technique for genetically elite donors often results in the recovery of immature oocytes. Because the chromosomes of immature oocytes are packed in a GV without a spindle apparatus, GV‐stage oocytes provide an alternative source of oocytes for MVC vitrification to avoid chromosomal misalignment due to microtubule depolymerization, which is observed in cryopreserved MII oocytes. The GV‐stage oocytes also avoid the possible risk of zona hardening due to the premature release of cortical granules. However, efficient cryopreservation of GV‐stage oocytes remains to be established, even for humans or small rodents (Brambillasca et al., 2013). Blastocyst yields from GV‐stage bovine oocytes after MVC vitrification are summarized in Table 2. The OPS tubing device or Cryotop surface device seems to be the most suitable for MVC vitrification of bovine immature oocytes.

**TABLE 2:** MVC vitrification of bovine immature (GV) oocytes surrounded with cumulus cells and blastocyst generation following IVM and IVF

Cryodevice (tubing [T]/surface [S])	CPA in VS	Blastocyst yield (%)^a^	Reference
OPS [T]	DMSO + EG + Suc	6	Vieira et al. (2002)
OPS [T]	DMSO + EG + Suc	4	Modina et al. (2004)
NM [S]	EG + Ficoll + Suc	8	Abe et al. (2005)
MD [device‐less]	DMSO + EG + Suc	2	Kim et al. (2007)
OPS [T]	EG + CD	18	Magnusson et al. (2008)
Cryotop [S]	DMSO + EG + Suc	11	Zhou et al. (2010)
MD [device‐less]	EG + Suc	8	Papis et al. (2013)
Cryotop [S]	DMSO + EG + Suc	3	Sprícigo et al. (2014)
Cryotop [S]	DMSO + EG + Suc	19	Ezoe et al. (2015)
OPS [T]	DMSO + EG + Suc	12	Yu et al. (2016)
Cryotop [S]	DMSO + EG + Suc	18	Tashima et al. (2017)
Cryotop [S]	EG + PG + PVP + Suc	15	Somfai and Hirao (2021)

Abbreviations: CD, cytochalasin‐D; CPA, cryoprotective agent; DMSO, dimethylsulfoxide; EG, ethylene glycol; MD, microdrop; NM, nylon mesh; OPS, open pulled straw; PG, propylene glycol; PVP, polyvinyl‐pyrrolidone; Suc, sucrose; VS, vitrification solution.

^a^ Blastocyst yields were calculated from the total number of cryopreserved oocytes.

**FIGURE 2:**
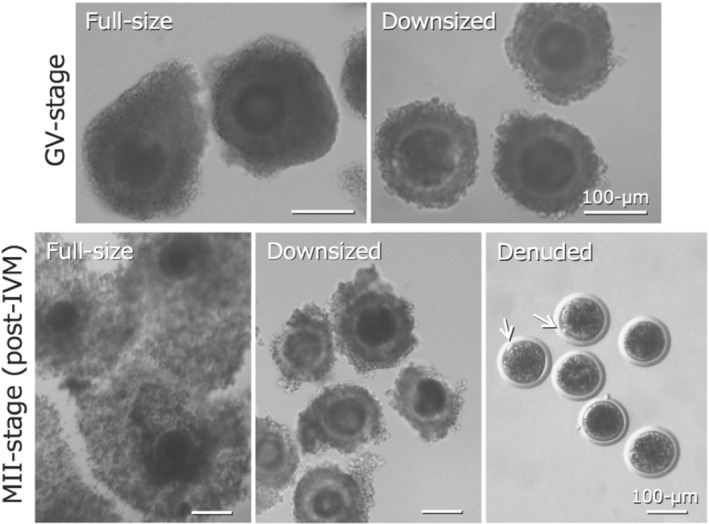
Bovine COCs before and after IVM. Immature GV‐stage oocytes are vitrified in the form of COCs, while mature MII‐stage oocytes can be vitrified after partial or full removal of cumulus layers (depending on the type of subsequent IVF system employed). The metaphase plate is concealed under ooplasmic lipid droplets, but the first polar body can be detected in the perivitelline space of fully denuded oocytes (arrows)

Bovine MII‐stage oocytes after ovulation or retrieved immediately before ovulation by ovum pick‐up or oocytes matured in vitro (IVM) have been cryopreserved with or without the surrounding cumulus cell layers (Figure 2). The GV‐stage oocytes are connected with the surrounding cumulus cell layers (cumulus‐oocyte complexes [COCs]) via gap junctions across the zona pellucida, involving proteins such as connexin‐37. Disruption of gap junction communications between the oocytes and surrounding cumulus cells is initiated immediately after IVM (Modina et al., 2004). The presence of several layers of cumulus cells in COCs may result in a delayed exchange between intracellular free water and permeating CPAs during equilibration and dilution phases of cryopreservation (Hyttel et al., 2000). Transmission EM observation indicated that hyperosmotic VS conditions and ultra‐rapid cooling during vitrification procedures had a detrimental impact on the functional integrity of gap junctions in domestic species (Fuku et al., 1995; Hochi et al., 1996). Some reports described the effect of full or partial denuding of bovine COCs on their cryotolerance (Figure 2). After Cryotop vitrification at the GV stage, Zhou et al. (2010) found 11% and 4% blastocyst yields from bovine full‐size COCs and partially denuded COCs, respectively. In contrast, we showed a positive effect of downsizing cumulus cell layers on cryotolerance after Cryotop vitrification, with 14%, 18%, and 8% blastocyst yields from bovine full‐size COCs, downsized COCs, and denuded oocytes, respectively (Tashima et al., 2017).

Ultra‐rapid cooling rates have been achieved by direct immersion of oocytes/cryodevice into cryogenic LN_2_ (−196°C) with a little boiling at first contact. A physical phenomenon “Leidenfrost effect,” which refers to the quick development of nitrogen gas bubbles, generates a pocket of nitrogen vapor around oocytes and results in delay of heat transfer through its insulator‐like action. Vitrification in N_2_ slush (a mixture of solid and liquid nitrogen) has been considered as a new strategy to increase the cooling rate of oocytes (Santos et al., 2012), because it can avoid the Leidenfrost effect. However, Martino et al. (1996), the pioneers of oocyte EM grid vitrification, reported a comparable or rather inferior blastocyst yield from bovine MII oocytes vitrified in N_2_ slush (−207°C) versus LN_2_ (−196°C). In OPS vitrification of bovine immature oocytes, one Chinese group published the suitability of liquid helium (−269°C) as an alternative to conventional LN_2_ (blastocyst yield 10% vs. 5%, Yu et al., 2016; 13% vs. 9%, Xu et al., 2017; 13% vs. 1%, Zhang et al., 2020). Interestingly, the immature oocytes vitrified in liquid helium had fewer intracytoplasmic lipid droplets after IVM compared with those vitrified in LN_2_ (Xu et al., 2017). Our laboratory also observed unique kinetics of intracytoplasmic lipid droplets (distribution and size variation) in bovine oocytes after the downsizing of cumulus layers, Cryotop vitrification, and IVM (Tashima et al., 2017). Another challenge for improving the cryotolerance of bovine immature oocytes is to eliminate DMSO from the VS, because this traditional CPA has a higher chemical toxicity than EG or propylene glycol (PG) (Awan et al., 2020). An equal proportion of EG and DMSO has been long considered the most efficient combination of permeating CPA in VS. The membrane permeability of DMSO is lower than that of EG, probably because of the involvement of different aquaporin channels (Edashige, 2016). In Cryotop vitrification of bovine immature oocytes, the use of EG/PG‐based VS instead of EG/DMSO‐based VS resulted in a higher IVM outcome (90% vs. 76%), but a comparable blastocyst yield (10% vs. 14% per matured oocytes; Faheem et al., 2015). Recently, Somfai and Hirao (2021) reported that the replacement of DMSO with PG in protein‐free VS with a few additional modifications resulted in a statistically comparable blastocyst yield from post‐warmed bovine immature oocytes (12% vs. 8%). In the above experiment, the positive effect of the EG/PG‐based protein‐free VS was clearly demonstrated in the total cell number of resulting blastocysts (118.1 vs. 56.5 cells).

## 5 CONCLUSIONS

This article focused on providing an up‐to‐date review of cryodevices used for MVC vitrification of bovine mature/immature oocytes in the past two decades, noting that a recent article has comprehensively summarized the bovine oocyte vitrification (Dujíčková et al., 2021). Various types of cryodevices (tubing‐type and surface‐type) have been developed to accelerate the cooling rate in oocyte MVC vitrification. A triangle NM sheet and HF with nano‐scale pores allowed the loading of large quantities of oocytes and the easy exchange of medium without complicated capillary operations. A multilayer SF sheet, another multiporous surface device, facilitated the minimization of oocyte‐surrounding VS volume through its high‐absorption property. Some of the cryodevices are commercially available as an open and/or closed system (e.g., Cryotop®/Cryotop®CL, CryoTip®, and Diamour‐op/Diamour‐cs). Cryodevices, such as OPS and Cryotop, have been used for the MVC vitrification of immature COCs with moderate or poor success, and await further cutting‐edge developments. Understanding and further improvement of cryodevice‐dependent performance for oocyte survivability will significantly contribute to efficient embryo production by IVM/IVF, in cattle breeding industries and also in human ART.

## CONFLICT OF INTEREST

The author declares no conflict of interest.
